# Hepatocyte nuclear factor 1 in renal lipid metabolism: molecular mechanisms and therapeutic potentials

**DOI:** 10.1007/s10565-025-10097-5

**Published:** 2025-11-26

**Authors:** Wenhui Zhu, Wenfan Wang, Yayun Wang, Xiaolin Tong, Xingfeng Liu, Lili Zhang, Linhua Zhao

**Affiliations:** 1 College of Traditional Chinese Medicine, Changchun University of Chinese Medicine, No. 1035, BoShuo Street, Changchun, 130117 China; 2 Institute of Metabolic Diseases, Academy of Chinese Medical Sciences, Guang’ Anmen Hospital, China, No.5, Beixiange Street, Xicheng District, Beijing, 100053 China; 3 Institute of Materia Medica, Chinese Academy of Medical Sciences and Peking Union Medical College, No.1, Xiannongtan Street, Xicheng District, Beijing, 100050 China; 4 The Affiliated Hospital of Changchun University of Chinese Medicine, No. 1478, GongNong Street, Changchun, 130117 China

**Keywords:** HNF-1, Renal lipid metabolism, Lipotoxicity, Kidney disease, Targeted therapy

## Abstract

Kidney disease is increasingly linked to dysregulated lipid metabolism, yet the molecular mechanisms driving renal lipotoxicity remain poorly understood. This review elucidates the pivotal role of the hepatic nuclear factor-1 family (HNF-1α and HNF-1β) in renal lipid homeostasis, integrating clinical and experimental evidence. Functionally, HNF-1 isoforms regulate lipid synthesis, oxidation, and transport via conserved POU domains and transcriptional networks. HNF-1α enhances high-density lipoprotein (HDL)-mediated cholesterol efflux through ApoM, while concurrently regulating PCSK9 to promote LDL receptor (LDLR) endocytosis and degradation, thereby inhibiting cholesterol uptake; whereas, HNF-1β promotes cholesterol synthesis via activation of HMGCR/SREBF2 and modulates the PCSK9-LDLR axis. Additionally, HNF-1β coordinates triglyceride metabolism through farnesoid X receptor (FXR) and peroxisome proliferator-activated receptor gamma (PPARγ) signaling pathways, and regulates mitochondrial fatty acid β-oxidation (FAO) via peroxisome proliferator-activated receptor gamma coactivator 1-alpha (PPARGC1A). Clinically, HNF-1α (MODY3) and HNF-1β (MODY5) mutations are closely associated with dyslipidemia, proteinuria, and CKD progression, with lipotoxicity serving as a key pathogenic driver. Therapeutic strategies targeting HNF-1 include pharmacological agents (e.g., metformin, GLP-1 agonists) and natural compounds (berberine, resveratrol) that modulate its transcriptional activity, alongside CRISPR and miRNA-based precision interventions. This review summarizes the important and multifaceted role of HNF-1 in renal metabolic disorders, highlighting its potential as a therapeutic target and offering new strategies for precision nephrology.

**Graphical Abstract:**

• The HNF-1 transcription factor regulates renal lipid metabolism by coordinating lipid synthesis, transport, and fatty acid oxidation.

• The HNF-1α/β maintains renal cholesterol homeostasis by regulating the HMGCR, PCSK9-LDLR, and ApoM pathways.

• HNF-1β deficiency impairs the PPARGC1A pathway, leading to dysfunction of fatty acid β-oxidation in the renal tubules.

• Targeting HNF-1 offers a novel precision therapy strategy for diabetic nephropathy.

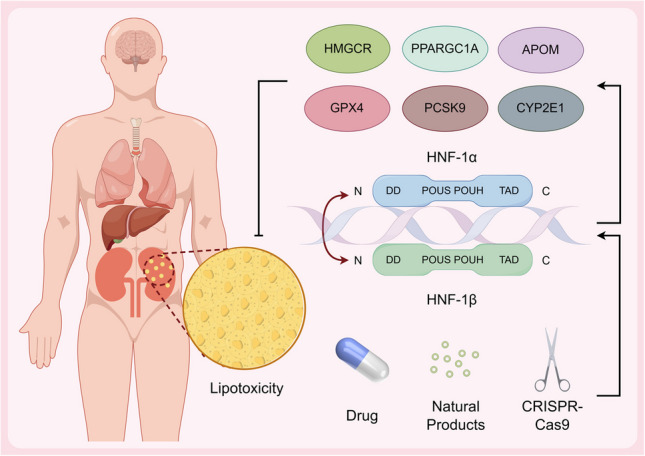

## Introduction

Amidst the escalating global burden of metabolic disorders, the pandemic prevalence of diabetes, obesity, and hypertension has substantially amplified the public health threat posed by kidney diseases(1, 2). Epidemiological data from the Global Burden of Disease Study 2021 (GBD 2021) reveal that the age-standardized global prevalence of chronic kidney disease (CKD) has reached 359 million cases, marking a 92% increase since 1990, with direct annual mortality exceeding 1.53 million(3, 4). Projections indicate renal diseases will emerge as the fifth leading cause of death worldwide by 2040(5). This alarming trajectory underscores the urgent need to elucidate molecular pathogenesis, particularly the central role of energy metabolism dysregulation in renal injury.

As a metabolically active organ, the kidney assumes pivotal roles in metabolic waste excretion, water-electrolyte homeostasis, and endocrine regulation, processes demanding substantial energy expenditure(6). Physiological investigations have revealed that renal ATP production predominantly originates from fatty acid β-oxidation (FAO), accounting for over 70% of total energy supply during fasting(7, 8). Intriguingly, when free fatty acid (FFA) influx surpasses mitochondrial oxidative capacity, excessive lipids accumulate in renal parenchymal cells through non-oxidative pathways. This pathological accumulation triggers a cascade of cellular disturbances, including endoplasmic reticulum (ER) stress, compromised mitophagy, and inflammasome activation, collectively termed lipotoxicity(9, 10). Since Rickards first reported the phenomenon of renal lipid deposition in 1883, this pathological feature has long been recognized as a concomitant alteration of metabolic disorders(11). Recent evidence suggests that localized lipid deposition in the kidney can act as a direct pathogenic factor independent of the systemic metabolic state, contributing to the core pathological processes of CKD-including glomerulosclerosis, programmed death of podocytes, and interstitial fibrosis-through lipotoxic mechanisms(12–15). Consequently, targeted modulation of the renal lipid metabolic network is a promising therapeutic strategy to mitigate the progression of chronic kidney disease.

The hepatocyte nuclear factor 1 (HNF-1) family, comprising two evolutionarily conserved isoforms (HNF-1α and HNF-1β), was originally identified as hepatic transcriptional regulators but has since been recognized as master coordinators of metabolic homeostasis across multiple organs including the liver, kidneys, and pancreas(16–18). As a key hub in the regulation of systemic lipid homeostasis, HNF-1 family exerts biological functions by coordinating the core pathways of lipid synthesis(19), oxidative metabolism(20, 21) and transport regulation(22). Accumulating evidence demonstrates that HNF-1α/β dysfunction is pathologically implicated in metabolic disorders, with well-characterized mechanistic roles in nonalcoholic fatty liver disease (NAFLD), obesity-associated lipotoxicity, and atherosclerotic plaque formation(23–25). Notably, clinical studies have revealed that dysregulated HNF-1 expression can lead to renal dysplasia and functional impairment(26) and significantly increase the risk of kidney-related diseases(27, 28). Nevertheless, despite the well-established association between HNF-1 and the renal system, critical gaps persist in understanding its molecular mechanisms governing renal lipid metabolism, particularly the subtype-specific regulatory networks and pathological transition mechanisms requiring elucidation.

In this review, we systematically describe the molecular structure and functional attributes of HNF-1, and summarize the divergent regulatory circuits of HNF-1α and HNF-1β isoforms in renal lipid metabolism. By synthesizing multimodal evidence from gene expression regulation, animal model experiments, and clinical case, we elucidate the regulatory mechanism of HNF-1 in renal lipid metabolism and revealed its dual role in lipotoxicity. Significantly, this review pioneers the systematic mapping of HNF1-targeted pharmacological landscapes, proposing modulation of the HNF-1 axis as an emerging therapeutic frontier for renal lipotoxicity. Our work bridges mechanistic gaps in renal metabolic research while establishing a rationale-driven roadmap for precision nephrology development.

## Hepatocyte nuclear factor 1 family

### Molecular structural and domain architecture of HNF-1

**Fig. 1:**
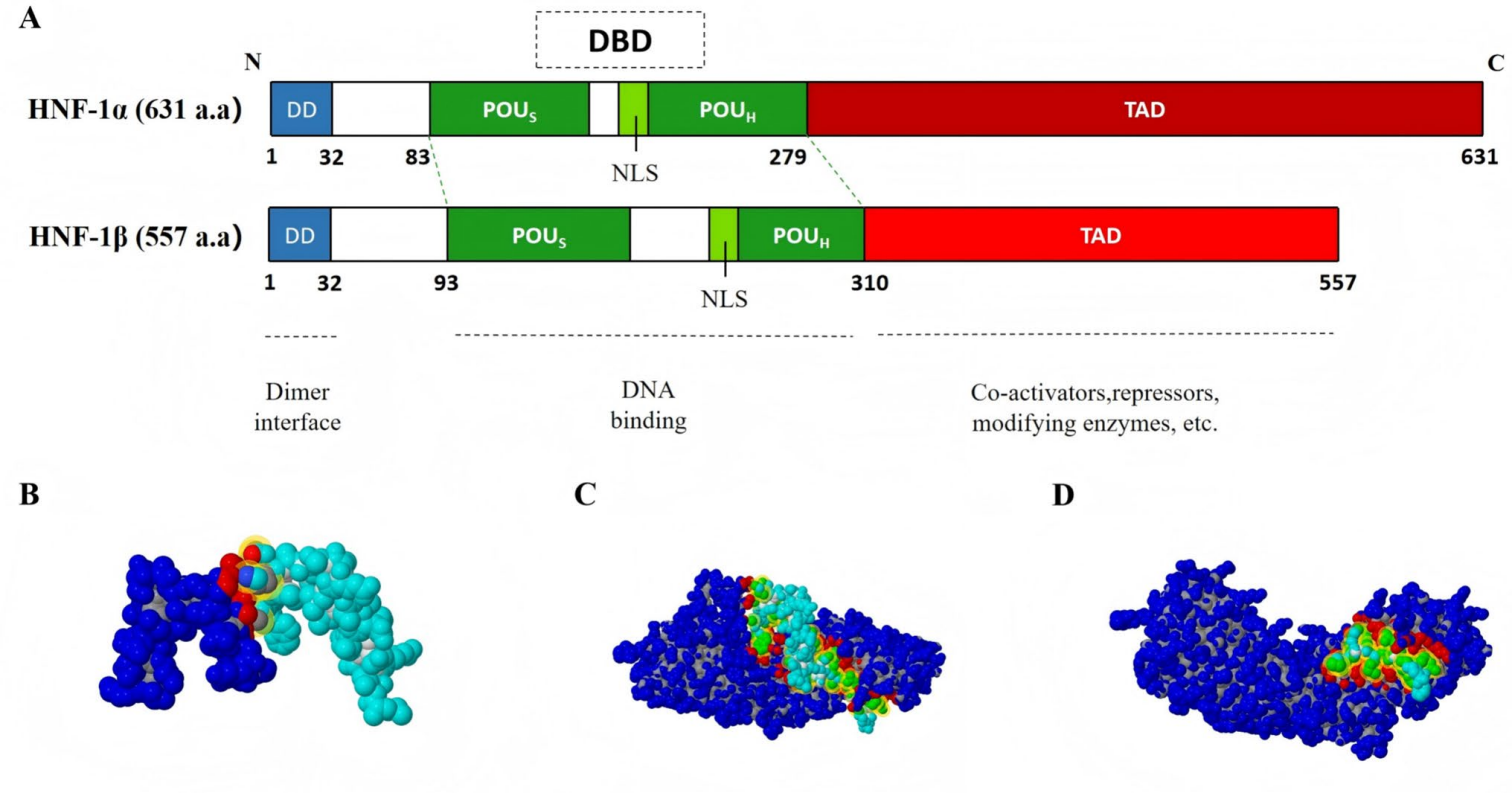
Molecular structure of the HNF-1 family. **A** Schematic diagram of the protein structural domains of HNF-1α/HNF-1β. **B** Stereo structure of the HNF-1α homodimer. **C** Stereo structure of HNF-1α and HNF-1β heterodimers. **D** Stereo structure of the HNF-1β homodimer. The polypeptide chains of human HNF-1α and HNF-1β consist of 631 amino acid residues and 553 amino acid residues, respectively, and can be classified into (i) N-terminal dimerization domain (DD); (ii) POUS domain for sequence-specific DNA recognition (POUS); (iii) POUH domain stabilizing DNA–protein interactions (POUH); and (iv) C-terminal transactivation domain coordinating coactivator recruitment (TAD). NSL: nuclear localization signal. Completed via PowerPoint

As a prototypical dimeric transcription factor, HNF-1 comprises two isoforms, *HNF-1α* and *HNF-1β*, which share over 70% amino acid sequence homology(29). Both isoforms assemble functional transcriptional complexes through homo- or heterodimerization mechanisms(30). Genome mapping analysis showed that *HNF-1α* is located at 12q24.31, while *HNF-1β* is located at 17q12(31, 32). Structurally, HNF-1α and HNF-1β are composed of 631 amino acid residues and 557 amino acid residues, respectively, and both contain four major structural domains: (i) N-terminal dimerization domain; (ii) POUS domain for sequence-specific DNA recognition; (iii) POUH domain stabilizing DNA–protein interactions; and (iv) C-terminal transactivation domain coordinating coactivator recruitment (C-TAD)(26, 33, 34) (Fig. 1). Mechanistically, the N-terminal dimerization domain governs both homodimeric and heterodimeric complex assembly, with differential oligomerization kinetics observed between isoforms(35). The dimerization co-factor of HNF-1 (DCoH) binds to the N-terminal domains of HNF-1α and HNF-1β, forming a unique four-helix bundle structure. This interaction stabilizes the dimeric conformation and enhances transcriptional activation efficiency through conformational remodeling(36, 37). The POUS domain consists of four α-helices, exhibiting structural similarity to the HTH motif of λ phage repressor. The third α-helix (Helix III) inserts into the DNA major groove, mediating sequence-specific binding to 5′-end motifs (e.g., ATGC/GTTA) through side chain interactions(38–40). The POUH domain adopts a canonical homeodomain fold with three α-helices, in which the third helix (recognition helix) forms hydrogen bonds and van der Waals interactions with 3′-end sequences (e.g., AAAT/ATTAAC) to stabilize binding(41). Collectively, the POUS and POUH domains constitute the classical POU-type DNA-binding domain. Their unique structural features and cooperative mechanism enable specific recognition of the inverted palindromic sequence 5′-GTTAATNATTAAC-3′(42–44). C-TAD regulates hydroxylation-dependent modifications and hydrophobic interaction network, which are key components in the transcriptional regulation of HNF-1 and important features for distinguishing the two isoforms(42, 45). Structural analyses reveal that HNF-1α's C-TAD contains multiple activation motifs that facilitate recruitment of histone acetyltransferases CBP/p300, thereby enhancing transcriptional efficiency through chromatin remodeling(46, 47). In contrast, HNF-1β exhibits attenuated transactivation potential, relying predominantly on cooperative interactions with ancillary factors like Zyxin and HDAC1(48, 49). Notably, *HNF-1β* truncation mutants lacking this domain exhibit abolished cofactor binding capacity, resulting in > 80% reduction in target gene expression—a finding underscoring the domain's indispensable role in preserving transcriptional complex integrity(50).

### Tissue specificity and developmental timing of HNF-1

The HNF-1 family members HNF-1α and HNF-1β, initially identified through sequence homology to α-protein(51), exhibit broad but distinct tissue distribution. Both isoforms are expressed in the kidney, liver, gallbladder, pancreas, colon, duodenum, stomach and lung(52), with notable differences: *HNF-1α* and *HNF-1β* showed opposite high and low expression in liver and lung, respectively(53). Co-expression occurs in the pancreas, gallbladder, and intestine, suggesting potential functional synergy in bile acid metabolism and intestinal barrier regulation. In renal tissue, immunofluorescence studies reveal compartment-specific localization—HNF-1α is restricted to the brush border of proximal tubules and distal tubules, whereas HNF-1β localizes to proximal tubules, distal tubules, and collecting duct epithelia(18, 54–56). This spatial divergence aligns with functional specialization: HNF-1α primarily regulates proximal tubular solute transport, while HNF-1β maintains glomerular filtration integrity and electrolyte balance(57, 58). Both isoforms are predominantly nuclear, though minor cytoplasmic pools are detectable. Developmentally, HNF-1β mRNA emerges earlier (mouse embryonic day 6.5) compared to HNF-1α, which is first detected in the yolk sac at embryonic day 8.5(59). Genetic ablation studies demonstrate divergent phenotypic consequences between HNF-1 isoforms: Targeted deletion of *HNF-1β* results in embryonic lethality at E6.5–7.0 due to defective differentiation of extraembryonic endoderm(60, 61), while mice with *HNF-1α* deficiency survive embryonic development but exhibit postnatal dysfunction in hepatic, pancreatic, and renal systems(17). These findings establish HNF-1β as indispensable for early organogenesis and highlight the role of HNF-1α in maintaining metabolic homeostasis.

### HNF-1 in systemic lipid regulation

HNF-1 functions as a pivotal transcription factor regulating lipid metabolism through multiple mechanisms. Its DNA-binding domain recognizes conserved sequences at promoters, introns, or distal regulatory elements(62), while the C-terminal transactivation domain recruits coactivators (e.g., CBP/p300) to form context-specific transcriptional complexes that modulate target gene expression(26, 63). HNF-1 plays a central role in systemic lipid homeostasis by transcriptionally regulating key metabolic regulators such as sterol regulatory element-binding protein 1c (SREBP-1c) and peroxisome proliferator-activated receptor γ (PPARγ)(19, 64). As the central hub of lipid metabolism, the liver is critically regulated by HNF-1. Clinical evidence demonstrates that the *HNF-1α* mutation (P291fsinsC) induces hepatic steatosis and localized inflammation, driving NAFLD progression(65). Similarly, *HNF-1β* mutations are strongly associated with dyslipidemia and hepatocellular dysfunction(66). In addition, pharmacological inhibition studies demonstrate that suppressing hepatic HNF-1 reduces cholesterol uptake, significantly alleviating lipid deposition and steatosis(67, 68). It is worth noting that the reduction in hepatic lipid uptake may lead to an increased retention of lipids in the bloodstream, contributing to systemic hyperlipidemia. Beyond hepatic regulation, HNF-1 dysregulation disrupts lipid metabolism in multiple organs, including heart(69, 70), pancreatic β-cell(71), intestine(72), and fat(73). Emerging evidence implicates HNF-1 dysregulation as a critical modulator of renal lipid homeostasis. A case study revealed that individuals with a deletion of the *HNF-1β* gene not only exhibited polycystic kidney disease and diabetes but also displayed prominent lipid metabolism disturbances, including severe hyperlipidemia and adipose tissue atrophy, highlighting the regulatory role of HNF-1β in renal lipid homeostasis(66). Although research on renal lipid metabolism remains limited, emerging studies have outlined the contours of HNF1-mediated regulatory networks, providing novel insights into its role in renal lipid metabolic regulation.

## Role of the HNF-1 in renal lipid metabolism

### HNF-1 dysfunction bridges dyslipidemia and nephropathy

Clinical and genetic evidence highlights the critical role of the HNF-1 gene family in lipid metabolism and renal function(74, 75). Patients with *HNF-1α* p. Gly292fs mutations exhibit elevated fasting non-esterified fatty acids (Carriers 621 μmol/L vs. non-carriers 441 μmol/L, p = 0.0039), impaired lipolytic suppression during oral glucose tolerance tests (OGTT; 117 μmol/L vs. 64 μmol/L, p = 3.1 × 10⁻5), and abnormal Body Mass Index (BMI) reduction(76). Among *HNF-1α* mutation carriers, 43.9% (25/57) develop microvascular complications (mild: 18; severe: 7) or microalbuminuria (10 cases)(77), while 36% progress to persistent proteinuria or chronic kidney failure with near-upper-limit total cholesterol levels (5.12 ± 0.18 mmol/L)(78). Mechanistically, HNF-1α is involved in substance reabsorption by regulating the expression of sodium-glucose cotransporter 2 (SGLT2) and sodium-phosphate cotransporter type IIa (NaPi-IIa)(17), and its dysfunction may drive systemic metabolic disturbances by disrupting the tubular lipid microenvironment.

A case study of a maturity-onset diabetes of the young type 5 (MODY5) patient with 17q12 microdeletion and *HNF-1β* mutation revealed severe dyslipidemia: TC (6.58 mmol/L), LDL (3.48 mmol/L), Apolipoprotein A1 (ApoA1)(2.02 g/L), and lipoprotein(a) (517 mg/L) elevation, accompanied by systemic lipoatrophy(66).Cohort analyses further demonstrated that HNF-1β/MODY5 patients commonly exhibited renal dysfunction, as evidenced by elevated blood urea nitrogen (19.2 ± 8.5 nmol/L), serum creatinine (380.1 ± 238.7 μmol/L), and uric acid levels (488 ± 30 μmol/L), concomitant with lipoprotein(a) concentrations at or above the upper normal limit(78). Lp(a)—a pro-inflammatory and pro-fibrotic particle composed of LDL-like cores and apolipoprotein(a)-fibrinogen-like domains(79)—may exacerbate renal injury through inflammatory pathways(80). A large cohort analysis revealed that the *HNF-1β* single nucleotide polymorphism (SNP) rs7501939-T allele positively correlates with VLDL subclass concentrations (P = 8.1 × 10⁻5–0.006) and inversely with large HDL particles (P = 0.004–0.038). However, these associations disappeared after adjusting for triglycerides (TG), suggesting that HNF-1β may indirectly influence lipoprotein profiles by modulating TG metabolism(81). Furthermore, multiple genome-wide association studies (GWAS) confirm links between the *HNF-1* locus and elevated total cholesterol, LDL-C, apolipoprotein B(82, 83), and rapid eGFR decline(84), underscoring its dual regulatory role in renal and lipid metabolism.

### Experimental evidence of HNF-1 orchestrates renal lipid

Clinical observations of renal-lipid comorbidities have been further validated in experimental models, revealing the regulatory roles of HNF-1 family members in renal lipid metabolism. In *HNF-1α* full knockout mice, sexual dimorphism is evident: female mice exhibit significantly higher serum triglycerides (579.5 mg/dL) compared to males (155.9 mg/dL), accompanied by with characteristic orange-yellow turbid blood lipids(85). This corroborates the disorders of lipid metabolism triggered by genetic defects. Further mechanistic investigations revealed that *HNF-1α*-deficient mice exhibited significantly increased urinary bile acid excretion accompanied by hypercholesterolemia(86), attributable to the complete absence of apical sodium-dependent bile acid transporter (ASBT) expression in kidneys and intestines, leading to impaired cholesterol metabolism(87). Notably, ASBT expression levels served as important prognostic indicators for clear cell renal cell carcinoma (ccRCC), a malignancy pathologically characterized by aberrant cholesterol accumulation(88, 89). These findings suggest that the HNF-1α/ASBT axis may play a key role in renal lipid dysregulation. In the adriamycin-induced nephropathy mouse model, we observed a marked reduction in renal mRNA levels of *HNF-1α* and *proprotein convertase subtilisin/kexin type 9 (PCSK9)*, concomitant with upregulated expression of low-density lipoprotein receptor (LDLR) and its transcriptional regulator SREBP-2. This transcriptional reprogramming mechanistically triggered enhanced renal cholesterol uptake and de novo synthesis. Notably, external inflammatory challenges were found to exacerbate this pathological cascade through synergistic modulation of lipid metabolic pathways(90).

Unlike HNF-1α, HNF-1β is essential during embryogenesis. Given that homozygous *HNF-1β* knockout mice result in embryonic lethality secondary to severe renal dysplasia, researchers have predominantly employed heterozygous models for mechanistic investigations(61). Notably, *HNF-1β* heterozygous mice carrying the p. Arg177Ter mutation demonstrated a 50% reduction (p < 0.05) in renal expression of long-chain fatty acid β-oxidation genes (CPT1α, ACADL) and glutathione metabolism-related transcripts(91). Additionally, Torell et al. reported *HNF-1β* mutant mice with selective renal accumulation of campesterol (fourfold increase, p < 0.05), accompanied by hepatic lipid dysregulation and impaired renal function(92). Campesterol reduces intestinal cholesterol absorption through competitive inhibition of Niemann-Pick C1-Like 1 (NPC1L1)(93), and its kidney-specific accumulation may reflect reprogramming of local cholesterol metabolic pathways. In addition to the mutant model, in a model of endotoxin-induced kidney injury, HNF-1β downregulation was able to directly inhibit the activity of the peroxisome proliferator-activated receptor gamma coactivator 1-alpha (PPARGC1A), which mediates the generation of lipid droplets, thereby impairing mitochondrial function and renal energy metabolism(94, 95).

Collectively, these animal studies provide evidence for the spatiotemporal regulatory specificity of HNF-1 family members in renal lipid metabolism, while in vitro experiments have helped to establish the direct mechanistic basis through their molecular interactions with downstream targets. In the Cos7 cell model, HNF-1α specifically activated the *apolipoprotein M (ApoM)* gene promoter in a dose-dependent manner, confirming its function as a core transcriptional regulator of the *ApoM* gene(96). The protein encoded by this gene is not only a core functional component of high-density lipoprotein (HDL), but also a key molecule mediating reverse cholesterol transport(97, 98). In renal tubular epithelia, HNF-1β exerts lipid metabolic control through mitochondrial regulation. The study by Piedrafita et al. demonstrated that *HNF-1β* ablation leads to severe mitochondrial dysfunction in proximal tubular cells, characterized by a 57% reduction in ATP production and a 66% decrease in fatty acid β-oxidation rates compared to wild-type controls (p < 0.001), alongside the accumulation of cytoplasmic lipid droplets. Mechanistically, HNF-1β directly binds to and activates the *PPARGC1A* promoter, thereby sustaining mitochondrial biogenesis and oxidative phosphorylation. Loss of *HNF-1β* disrupts this regulatory axis, resulting in *PPARGC1A* suppression and compromised hypoxic stress adaptation(94, 99). Chromatin immunoprecipitation followed by sequencing (ChIP-seq) and transcriptomic profiling by Karam et al. systematically identified 1,545 protein-coding genes under direct *HNF-1β* regulation in murine renal epithelium, including key cholesterol regulators *3-hydroxy-3-methylglutaryl-CoA reductase* (*HMGCR)* and *PCSK9*(100). These results emphasize the important role of HNF-1 on renal lipid metabolism.

## Potential mechanisms of HNF-1 regulation of renal lipid metabolism

HNF-1, as a core transcriptional regulator of metabolic homeostasis, achieves multi-layered physiological regulation through its unique domain architecture. This protein forms homodimers or heterodimers and specifically recognizes the palindromic sequence GTTAATNATTAAC in target gene promoters via its POU domain, thereby activating downstream gene transcription(22, 62). Its precise DNA-binding and transactivation capabilities establish it as a key regulatory hub for lipid metabolism across tissues, particularly in the liver and kidney systems. In hepatic metabolism, the regulatory mechanisms of HNF-1 in pathways such as lipid synthesis, uptake, transport, storage, and fatty acid oxidation have been relatively well-defined(101). In contrast, significant gaps remain in understanding its role in renal cholesterol metabolism, triglyceride homeostasis, and fatty acid oxidation. Current evidence suggests that HNF-1 may participate in renal lipid homeostasis through multiple pathways, but the specific molecular mechanisms require systematic investigation (Table 1).

**Table 1:** Regulatory pathways of HNF-1 in renal lipid metabolism

HNF1 intervention	In vivo*/vitro*	Model	Targets	Binding site	Lipid metabolism	References
HNF-1β ↓	In vivo* vitro*	mIMCD3、Hnf-1β^−/−^ mice	HMGCR↓ SREBF2↓	-	Cholesterol synthesis↓	(100)
HNF-1β ↓	In vivo* vitro*	mIMCD3、Hnf-1β-/- mice	PCSK9↓ LDLR↑	419 bp upstream from the start codon	Cholesterol intake↑	(100)
HNF-1α ↓	In vivo	BALB/c mice	PCSK9↓ LDLR↑	28 bp upstream of promoter	Cholesterol intake↑	(90)
HNF-1α ↓	In vivo* vitro*	Hnf-1α^⁻/⁻^mice Hnf-1α^⁺/⁻^mice Cos7 cells	ApoM↓	88 to 103 upstream of promoter	Cholesterol transport↓	(96)
HNF-1β	In vivo* vitro*	Hnf-1β^f/f^ mice、mIMCD3	FXR↓	-	-	(102)
-	In vivo	C57BL/6 mice	FXR↓ SREBP-1c↑	-	Lipid synthesis↑	(103)
-	In vitro	PTECs	FXR↑ PPARγ↑	-	FAO↑	(104)
HNF-1β	In vitro	LLC-PK cells	G6Pase	231 to 199 upstream of promoter	-	(105)
-	In vivo	K-G6pc-/- mice	G6Pase↓ ChREBP↑ FASN↑ ACC↑	-	Lipid synthesis↑	(106)
HNF-1α	In vivo* vitro*	LLC-PK1 cells、HNF-1α- Mut mice	PCK1	200 to 180 upstream of promoter	-	(107)
-	In vivo* vitro*	PTs、Pck1 ^−/−^ mice	PCK1↓	-	Mitochondrial function↓	(108)
HNF-1α ↓	In vivo	LPS-induced mice	CYP2E1↓	-	ROS↓ lipid peroxidation↓	(109)
HNF-1β ↓	In vivo* vitro*	C57Bl6 mice、HK-2、MCT cells	PPARGC1A↓	4171-bp upstream of the transcription site	Fatty acids β-oxidation↓	(94) (99)
HNF-1β ↓	In vitro	MCCs、HEK293T cells	ACSL4↑ GPX4↓	-	ROS↑ lipid peroxidation↑	(110)
HNF-1β ↑	In vitro	HEK293	ACE2↑	-	-	(111)
-	In vivo* vitro*	C57/BL6 mice、PTC	ACE2↑ Nrf2↓	-	Lipid synthesis↓	(112)

### The potential regulatory mechanism of HNF-1 in renal cholesterol metabolism

The HNF-1 family regulates renal cholesterol metabolism through multiple pathways, primarily modulating cholesterol synthesis, uptake, and transport. In the regulation of cholesterol synthesis, HNF-1β critically maintains renal cholesterol synthesis by directly modulating transcriptional activity of *HMGCR*, the rate-limiting enzyme in cholesterol biosynthesis, and *sterol regulatory element-binding transcription factor 2 (SREBF2)*, a master cholesterol homeostasis regulator(100, 113, 114).In dominant-negative *HNF1-β* mutants lacking the C-TAD renal epithelial cells and *Hnf-1β *^*−/−*^ mice, transcriptional suppression of *HMGCR* and *SREBF2* reduces cholesterol synthesis rates by 55%, accompanied by diminished intermediates such as 7-dehydrocholesterol (7-DHC)(100, 115). Notably, systemic cholesterol remained stable despite limited local renal cholesterol synthesis, suggesting that renal cholesterol synthesis is not a major source of circulating cholesterol. In addition, HNF-1likewise plays an important role in cholesterol uptake.

HNF-1 regulates LDLR-mediated cholesterol uptake via PCSK9 transcriptional control. HNF-1β directly activates a conserved cis-element 419 bp upstream of the *PCSK9* exon 1, enhancing promoter activity 3.5-fold, while binding-site mutations reduce activity by 50%(100). In inflammatory models, coordinated reductions in renal HNF1-α and PCSK9 mRNA/protein levels correlate with elevated LDLR expression, driving cholesterol accumulation(90). In terms of molecular mechanisms, HNF-1 transcriptionally regulates renal PCSK9 expression and promotes cell surface LDLR lysosomal degradation pathway, which in turn enables precise regulation of renal cholesterol uptake capacity(116).These studies revealed the specific regulation of renal cholesterol metabolism by HNF-1: on the one hand, it promotes cholesterol synthesis through activation of the HMGCR/SREBF2 axis, and on the other hand, it maintains renal cholesterol metabolism homeostasis by inhibiting LDLR-mediated cholesterol uptake through up-regulation of PCSK9.

In addition, HNF-1 exhibits a unique regulatory function in cholesterol transport. HNF-1α transcriptionally activates *ApoM* by binding an evolutionarily conserved promoter region (−103 to −88 bp). *HNF-1α*^*−/−*^ mice exhibit complete renal ApoM loss, with heterozygotes showing dose-dependent serum ApoM reductions. Electrophoretic mobility shift assay (EMSA) and luciferase reports, verifying that HNF-1α directly binds and activates the *ApoM* promoter(96). As ApoM is a key component of HDL, HNF-1α protects renal health by enhancing the efficiency of HDL-mediated cholesterol efflux(96, 117, 118). On this basis, the factor can simultaneously coordinate the inhibition of LDLR-mediated cholesterol uptake, thereby limiting the uptake of circulating cholesterol. This coordinated regulation suggests the existence of a multidimensional protective system that simultaneously promotes cholesterol export and limits exogenous cholesterol uptake, which may represent a novel mechanism for renal lipotoxicity protection.

### Potential molecular mechanisms by HNF-1 coordinates renal triglyceride metabolism

As a central regulatory factor in renal lipid metabolism, the HNF-1 family not only coordinates cholesterol homeostasis but also integrates triglyceride metabolism through farnesoid X receptor (FXR) signaling, thereby achieving comprehensive regulation of multi-lipid systems. In renal pathologies, FXR and its target genes exhibit significant dysregulation, correlating with lipid accumulation(119). HNF-1α directly binds a conserved *FXR* promoter region (–201 to –217 bp), enhancing its activity by sixfold, as validated by EMSA and luciferase assays(86). Although this phenomenon was initially identified in the liver, subsequent studies have shown that HNF-1β has a similar regulatory function in the kidney. Specifically, *HNF-1β* mutant mice exhibit significant downregulation of FXR expression in kidneys, while under hyperosmotic stress conditions, HNF-1β directly binds to and transcriptionally activates the *FXR* promoter(102). In type 1 diabetic models, *FXR* deficiency triggers aberrant activation of SREBP-1c and its downstream lipogenic targets (FASN, ACC, SCD-1), while simultaneously upregulating LDL receptor and lectin-like oxidized low-density lipoprotein receptor 1 (LOX-1) expression(103). This dual dysregulation synergistically amplifies renal lipid anabolism and ectopic uptake, exacerbating lipotoxic stress in diabetic nephropathy. Notably, FXR agonist treatment effectively ameliorates the aforementioned pathological processes. In the renal tissue of *db/db* mice, FXR agonist suppresses SREBP-1c expression while enhancing transcriptional activity of ATP-binding cassette sub-family A member 1 (ABCA1), a key cholesterol efflux regulator(120–122). This dual-targeted intervention significantly reduces renal triglyceride accumulation, yet exerts limited regulatory effects on intrarenal cholesterol homeostasis. New research demonstrates that FXR activation enhances FAO in renal proximal tubular epithelial cells via PPARγ-mediated signaling, with FXR overexpression models exhibiting increased enzymatic activity of carnitine palmitoyltransferase 1α (CPT1α)—the rate-limiting enzyme in mitochondrial β-oxidation—alongside reduced intracellular lipid droplet accumulation(104). Furthermore, HNF-1 is a core transcriptional regulator of Glucose-6-phosphatase (G6Pase), with tissue-specific mechanisms. In the liver, HNF-1 is co-regulated with *CREB1/CREB2* to accomplish the regulation of *G6Pase*; whereas in the kidney, HNF1-β dominates the activation of *G6Pase* by cAMP/PKA signaling through binding to its promoter region response element (–221 to –209 bp)(105). Clar et al. revealed that renal-specific *G6Pase* deficiency in mice triggers pathological glucose-6-phosphate (G6P) accumulation, which aberrantly activates carbohydrate response element-binding protein and its downstream lipogenic targets, driving de novo lipogenesis and culminating in elevated triglyceride biosynthesis within the renal cortex(106, 123).

### Potential modulation of renal fatty acid metabolism by HNF-1

The HNF-1 transcription factor family exerts multidimensional regulatory effects on renal fatty acid metabolism and oxidative stress. In renal tubular epithelial cells, HNF-1α directly binds the *phosphoenolpyruvate carboxykinase 1 (PCK1)* promoter region (–200 to –180 bp). Mutations at this binding site reduce basal PCK1 transcriptional activity by ~ 50% and impair nuclear receptor (e.g., PPARα, RXR) co-regulatory effects(107, 124). Clinically, patients with *PCK1*-deficiency develop significant abnormal fat accumulation in the liver and kidneys, which may be associated with disturbances in mitochondrial energy metabolism triggered by deficiencies in the function of key enzymes in the gluconeogenic pathway(125). Furthermore, in diabetic nephropathy, PCK1 overexpression mitigates renal fibrosis, preserves mitochondrial function, and reduces lipotoxicity-associated tubular apoptosis(108, 126). Functioning as a key mediator of lipid peroxidation(127), *cytochrome P450 2E1 (CYP2E1)* is transcriptionally activated in the liver via HNF1α-dependent binding to its promoter region(128, 129). In AKI mouse models, Cheng et al. replicated this regulatory axis using co-immunoprecipitation (Co-IP) and chromatin immunoprecipitation (ChIP) assays, demonstrating that inhibition of HNF-1α reduces renal *CYP2E1* transcriptional expression, thereby inhibiting renal reactive oxygen species (ROS) production and lipid peroxidation(109). These findings implicate HNF-1α in renal lipotoxicity pathogenesis. Intriguingly, despite the opposing roles of PCK1 and CYP2E1 in renal fatty acid metabolism, HNF-1α orchestrates their activities via promoter-specific transcriptional regulation. This bidirectional control mechanism may underlie HNF-1α's dualistic role in balancing renal lipotoxic stress.

**Fig. 2:**
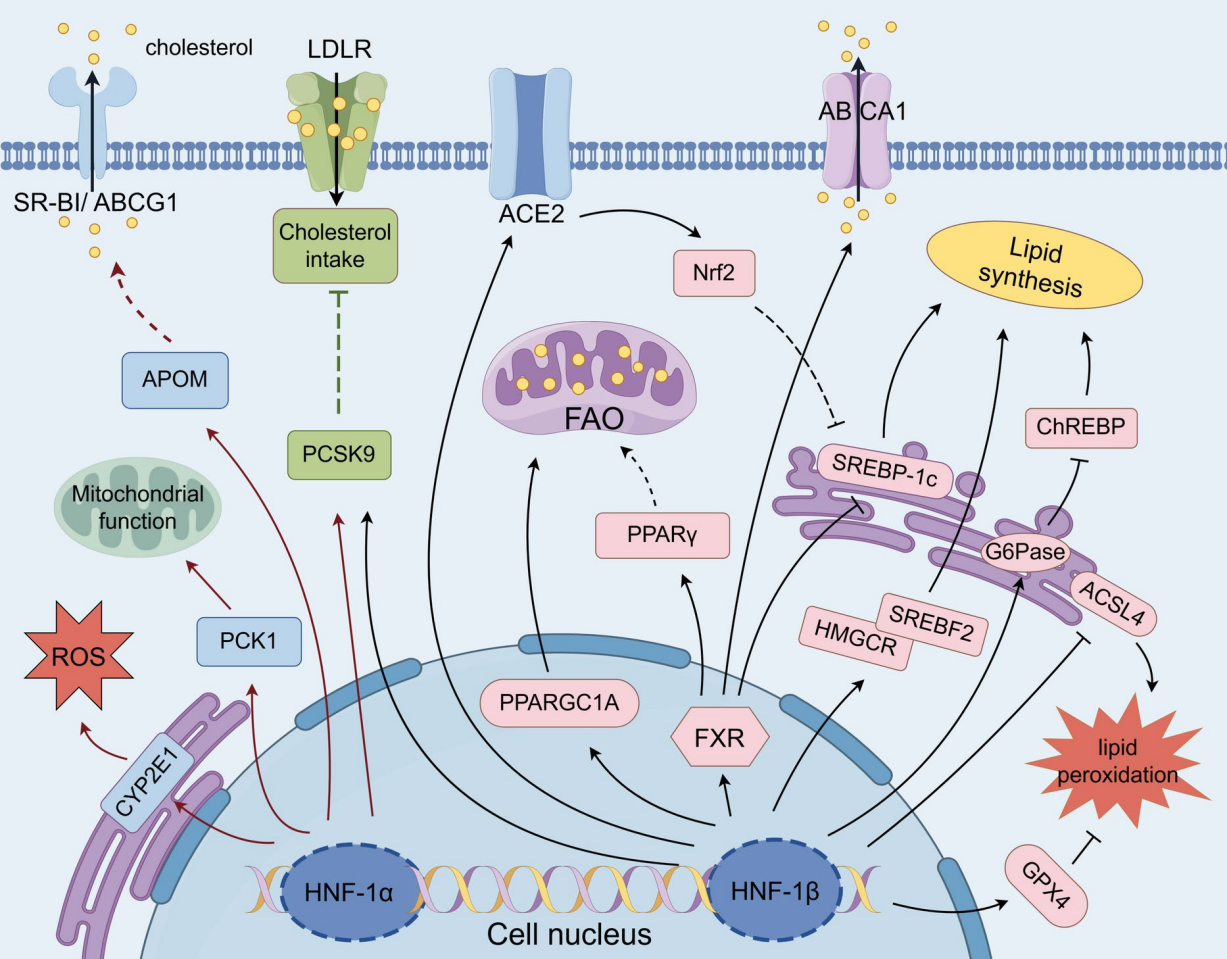
The potential regulatory roles and pathways of HNF-1 in renal lipid metabolism. HNF-1α/β mediates renal pathological alterations by regulating renal cholesterol synthesis and transport, lipid uptake, and fatty acid β-oxidation. Solid lines indicate direct relationships; dashed lines indicate indirect relationships. Arrows indicate promotion; flat heads indicate inhibition. Yellow particles represent lipids. Created by FIGDRAW (https://www.figdraw.com)

The report shows that HNF-1β directly regulates *PPARGC1A* transcription, governing proximal tubule mitochondrial morphology and respiration(94). In its defective cells, down-regulation of *PPARGC1A* transcription resulted in impaired cellular oxidative phosphorylation and decreased fatty acid oxidative utilization, and aberrant intracellular lipid accumulation(99). Furthermore, HNF-1β dysfunction disrupts the regulatory balance of ferroptosis. Transfection of a plasmid harboring the *HNF-1β* mutation (c.445C > A) into mouse renal mesangial cells significantly up-regulated the expression level of the promoter of ferroptosis, *acyl-CoA synthetase long-chain family member 4 (ACSL4)* and down-regulated the expression level of the ferroptosis protector, *glutathione peroxidase 4 (GPX4)* expression levels, while leading to increased intracellular ROS levels, which enhanced cellular sensitivity to iron death and exacerbated lipid peroxidation processes(110). Sabine et al. showed that HNF-1β can regulate the transcription of *angiotensin-converting enzyme 2 (ACE2)* by directly binding to HNF1-specific sites in its promoter region(111). In the *ACE2*-deficient ORG mouse model, the researchers observed an upregulation of the expression of lipid synthesis genes (ADRP, ACC, FASN), accompanied by the inactivation of the Nuclear factor erythroid 2-related factor 2 (Nrf2) signaling pathway, which was manifested as an abnormal deposition of renal lipids(112). Further studies revealed that ACE2 overexpression ameliorated lipid deposition by activating the Nrf2 pathway through a mechanism involving Nrf2-mediated down-regulation of lipid synthesizing factors (FASN, ACC, SREBP-1c), which reduces the overproduction of FFA and triglycerides (TG)(112, 130). Notably, the amelioration of lipotoxicity via this mechanism is often accompanied by the attenuation of other injury phenotypes, such as inflammation and renal fibrosis. HNF-1 orchestrates renal lipid metabolism through dynamic regulation of synthesis-degradation balance and oxidative stress responses. Dysregulation of this network triggers lipid synthesis/oxidation imbalance, exacerbating lipotoxic injury. In summary, targeting the HNF1-mediated pathways of cholesterol synthesis, lipid uptake, and mitochondrial oxidation can effectively attenuate lipotoxicity-driven renal pathological injury (Fig. 2).

## HNF-1 clinical translation and targeted therapy

### Clinical associations and translational applications

The elucidation of HNF1-mediated molecular networks not only underscores its central role in renal lipid metabolism but also highlights its clinical relevance and translational application value. Genetic mutations and dysregulation of the HNF-1 family represent critical etiological factors in renal developmental anomalies(131) and metabolic disorders(132). In maturity-onset diabetes of the young (MODY), *HNF-1α* mutations cause MODY3 (OMIM #600,496)(133), while *HNF-1β* mutations lead to MODY5 (OMIM #137,920)(134), both strongly associated with renal lipid dysregulation(135). Clinically, 61–90% of *HNF-1β* mutation carriers exhibit structural or functional renal abnormalities(136), and their pathological phenotypes show significant heterogeneity, mainly covering renal ciliopathy with anomalies of the kidney and urinary tract (RCAD)(26), glomerulocystic kidney disease (GCKD)(137), autosomal dominant tubulointerstitial kidney disease (ADTKD)(138), and congenital anomalies of the kidney and urinary tract (CAKUT)(139). Notably, 12% of *HNF-1β* mutation carriers progress to end-stage renal disease (ESRD)(131, 140), with renal functional decline potentially linked to systemic and intrarenal lipid accumulation. These patients frequently exhibit dyslipidemia, characterized by elevated non-esterified fatty acids (NEFA)(76), abnormal total cholesterol(78), and increased apolipoproteins(66).

Currently, the clinical association between HNF-1 family abnormalities and renal diseases has been gradually translated from molecular mechanisms to diagnostic applications. At the histopathological level, the immunohistochemical deletion of HNF-1β protein has emerged as a critical pathological criterion for distinguishing chromophobe renal cell carcinoma (ChRCC) from renal oncocytoma (RO), and its diagnostic efficacy is significantly better than that of traditional morphologic indicators(141). In the field of liquid biopsy, technology development based on exosomal assays is rapidly advancing. It has become possible to detect a potential correlation between HNF-1β mRNA levels in urinary exosomes and the renal tubular injury marker KIM-1/NGAL(142), the dynamics of which may reflect the progression of renal decline (multicenter data validation is required)(143). In addition, non-invasive prenatal testing techniques have begun to explore the association between *HNF-1β* mutations and congenital kidney malformations, and their technical feasibility has been validated in other single-gene renal disorders, such as the polycystic kidney disease (PKD)(144). In the future, a standardized testing process needs to be established through multicenter cohorts, and a multimodal diagnostic model integrating genomic (HNF-1), metabolomic (lipid profiles), and imaging histological features needs to be constructed to ultimately establish a full-cycle management system from risk warning to prognostic assessment.

### Enlightenment of HNF1-targeted interventions for renal lipid metabolism

HNF-1, as a core transcription factor in metabolic regulation, its aberrant expression or modification is closely related to diabetes mellitus, obesity, non-alcoholic fatty liver disease, and atherosclerosis. In recent years, targeted interventions against HNF-1 have demonstrated substantial progress in the fields of drug retargeting, bioactive natural compound development and epigenetic regulatory mechanisms. These results not only provide new ideas for the treatment of metabolic diseases, but also provide important insights for exploring the mechanism of action and intervention of HNF-1 in renal pathology, as summarized in Table 2.

**Table 2:** Drug-Mediated Regulation of HNF-1α/HNF-1β in Lipid Homeostasis

Drugs	HNF-1 Regulation	Model	Key Targets/Pathways	Functional Outcome	References
Metformin	HNF-1α ↓	HepG2 cells、HuH7 cells	AMPK phosphorylation、SREBP-2、PCSK9	Reduces circulating LDL-C levels	(145)
Liraglutide	HNF-1α ↓	HepG2 cells、db/db mice	PCSK9、LDLR	Reduces blood lipid levels 、 Improvement of liver lipid accumulation	(146)
Pravastatin	HNF-1α ↓	Wistar rats	LDLR、ACAT2、LXR、 PPARα、SREBP-2	Reduces liver cholesterol levels	(147)
Lunasin	HNF-1α ↓	HepG2 cells、ApoE^−/−^ mice	PCSK9、LDLR	Reduces plasma TC and LDL-C concentrations	(148)
leptin	HNF-1α ↑	HepG2 cells	PCSK9 、LDLR、p38-MAPK	Reduces LDLR levels in HepG2	(149)
Berberine	HNF-1α ↓	HepG2 cells、HEK293 cells、FVB mice	PCSK9、LDLR、Ubiquitin–Proteasome Degradation Pathway	Reduces plasma LDL-C leves	(150)
Resveratrol	HNF-1β ↑	C57BL/6 mice	GPX1	Improvement of glucose-lipid metabolism	(151)
Polygonatum odoratum	HNF-1β ↑	THP-1 macrophages、LDLR^−/−^mice、ApoE^−/−^mice	Sortilin	Inhibits aortic lipid deposition	(152)
curcumin	HNF-1α ↓	Syrian Golden Hamsters、Caco-2 cells、 HepG2 cells	SREBP-2、NPC1L1	Reduces intestinal and liver cholesterol absorption	(68)
Lycopene	HNF-1α ↓	ApoE^−/−^mice	NPC1L1	Inhibits intestinal cholesterol absorption and atherosclerosis	(153)
Oleanolic acid	HNF-1β ↑	C57BL/6 mice、3T3-L1 cells	NOX4、PPARγ、GPX1	Reduces oxidative injury and glucose/lipid metabolism dysfunction	(154)
Qushihuayu	HNF-1α ↑	Wistar rats	HNF-4α、FOXA3	Improves liver reprogramming and fibrosis	(155)

The classic antidiabetic drug metformin enhances AMP-activated protein kinase (AMPK) phosphorylation, modulating transcriptional activities of *HNF-1α* and *SREBP-2*, thereby suppressing hepatic PCSK9 expression and promoting low-density lipoprotein cholesterol (LDL-C) clearance(145). Similarly, the glucagon-like peptide-1 (GLP-1) receptor agonist liraglutide reduces PCSK9 levels in an HNF1α-dependent manner, ameliorating lipid metabolism disorders in type 2 diabetes(146). Pravastatin restores bile acid and cholesterol homeostasis in chronic cholestatic rats by downregulating HNF-1α(147), while the natural potentiator Lunasin antagonized the HNF1α-PCSK9 signaling axis, enhancing TG and LDL-C reductions by more than 50% with simvastatin(148). Leptin antagonists further reverse LDLR suppression by targeting the HNF-1α/PCSK9 pathway, restoring lipid clearance capacity(149). These investigations can give us the insight that lipotoxic injury in diabetic nephropathy can be alleviated by targeting renal HNF-1α and modulating the PCSK9-LDLR pathway.

Natural products demonstrate significant therapeutic potential in modulating HNF1-mediated pathways. Berberine degrades HNF-1α protein via the ubiquitin–proteasome system, suppressing *PCSK9* transcription and reducing serum total cholesterol levels(150). Resveratrol activates the HNF1-β/GPX1 pathway to mitigate hexavalent chromium [Cr (VI)]-induced glycolipid metabolic disturbances(151), indicating that it may attenuate heavy metal nephrotoxicity through renal oxidative stress mitigation and lipid metabolic reprogramming. Polygonatum flavonoids enhance HNF1-β SUMOylation, inhibiting sortilin-mediated lipid accumulation and delaying atherosclerotic plaque formation(152), and this post-translational modification modulation pattern may be applicable to the repair of protein stability in *HNF1β*-mutant polycystic kidney disease. Both curcumin(68) and lycopene(153) were able to reduce intestinal cholesterol absorption by inhibiting the SREBP-2/HNF-1α axis or down-regulating the expression of NPC1L1 via HNF-1α, suggesting that HNF-1 may influence systemic lipid metabolism by regulating the intestinal-renal axis, providing a new target for intervening in renal lipid disorders. Oleanolic acid reverses polychlorinated biphenyl (PCB)-induced oxidative stress and insulin resistance through the HNF1β-PPARγ signaling pathway(154), with its antifibrotic effects warranting validation in renal interstitial fibrosis models. Additionally, the herbal compound QushiHuayu upregulates HNF-1α/HNF-4α/FOXA3 expression, promoting hepatic stellate cell (HSC) reprogramming to ameliorate non-alcoholic steatohepatitis (NASH)(155). Its active components may offer candidates for treating renal interstitial fibrosis.

Non-coding RNAs (ncRNAs) and post-translational modifiers offer innovative strategies for targeting HNF-1. miR-802 blocks glycolysis-dependent ovarian clear cell carcinoma by inhibiting *HNF-1β* expression(156). miR-92a slows PKD progression by binding to the 3' untranslated region (3'UTR) of *HNF-1β*(157). miR-92a is an HNF-1β inhibitor which stabilizes its expression by blocking HNF-1α ubiquitination and degradation stabilizes its expression(158). These findings highlight the potential of renal-specific gene-editing tools, such as CRISPR activation systems or siRNA delivery platforms, for precision modulation of HNF-1 pathways in kidney diseases. In summary, the multi-organ targeting strategies for HNF-1 provide multidimensional paradigms for renal disease research: extending classical drug mechanisms, multi-target interventions using natural products, and precision adaptation of gene-editing tools, collectively revealing therapeutic potential in renal lipid metabolism. Future efforts should integrate multi-omics data with interdisciplinary technologies to advance HNF1-targeted therapies toward clinical precision medicine.

## Conclusions, limitations, and future perspectives

**Fig. 3:**
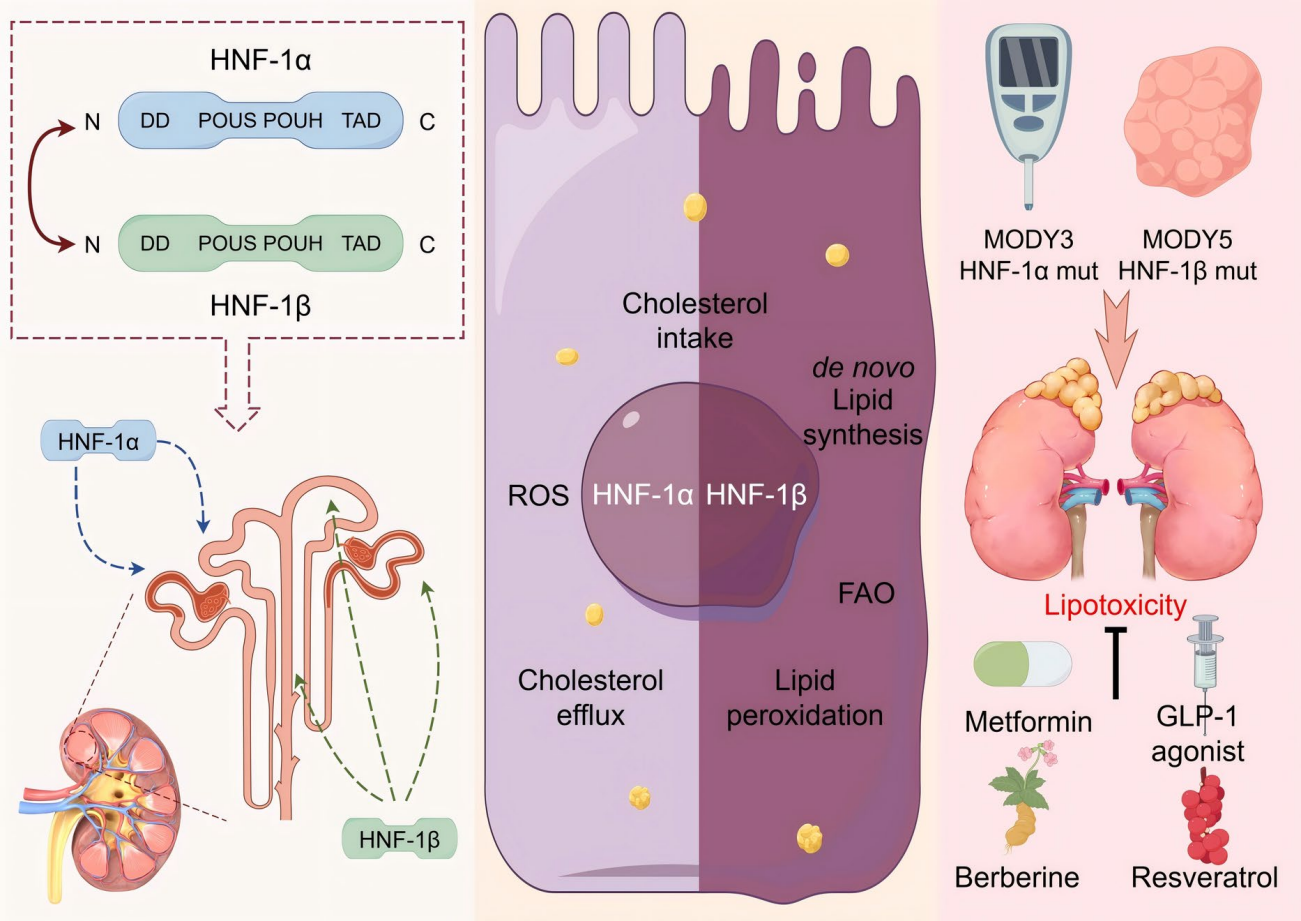
The HNF1 family is a potential therapeutic target for the regulation of renal lipid metabolism. Left: HNF-1 dimer structure and localization in renal tubules. Center: HNF-1 regulates cholesterol uptake and efflux, lipid synthesis, fatty acid oxidation (FAO), reactive oxygen species (ROS), and lipid peroxidation in the kidney. Right: HNF-1α/β mutations cause MODY3/5, leading to renal dysfunction; various drugs and natural products alleviate lipid metabolism disorders in the kidney caused by HNF-1α/β abnormalities. Created by FIGDRAW (https://www.figdraw.com)

The HNF-1 family (HNF-1α and HNF-1β) acts as a potential regulator of renal lipid metabolism by binding conserved promoter sequences (e.g., GTTAATNATTAAC) through their POU domains, dynamically coordinating cholesterol, triglyceride, and fatty acid metabolic networks (Fig. 3). However, their regulatory roles exhibit dualistic complexity. For instance, HNF-1β activates HMGCR/SREBF2 to promote cholesterol synthesis while simultaneously upregulating PCSK9 to suppress LDLR-mediated cholesterol uptake. It also enhances mitochondrial β-oxidation via PPARGC1A activation, creating a dynamic "synthesis-clearance" balance. This paradoxical mechanism likely reflects its precise control of lipid homeostasis—maintaining baseline metabolic demands while preventing pathological lipid overload. Under chronic inflammatory conditions, dysregulation of the PCSK9-LDLR axis may tip this balance toward excessive cholesterol synthesis, exacerbating renal lipid deposition. Similarly, HNF-1α reduces lipid accumulation by promoting LDLR degradation and improves mitochondrial function through PCK1 upregulation. Yet, its induction of CYP2E1 may aggravate renal tubular injury via ROS overproduction, highlighting the context-dependent nature of its effects.

Although clinical and mechanistic studies have established a robust association between HNF-1 family dysfunction and renal/lipid metabolic disorders, and existing pharmacological interventions (e.g., pravastatin, berberine) have shown preliminary results in improving lipid homeostasis by targeting HNF-1α/β, multiple limitations and challenges remain. First, the existing evidence base exhibits model-dependent heterogeneity. Substantial mechanistic data in the current literature originate from systemic or conditional gene knockout mouse models or acute toxin-induced injury models. While these models are crucial, they may not fully replicate the slow, multifactorial pathogenesis of human diabetic nephropathy or obesity-associated glomerulopathy. Furthermore, the observed differences in lipid metabolism phenotypes across distinct models suggest that HNF-1 signaling may depend on the microenvironmental context of specific models. Its functional regulatory mechanisms, particularly post-translational modifications, warrant further investigation. Second, there is a predominant reliance on correlative rather than direct causal evidence in adult renal pathologies. For instance, while HNF-1β deletion is linked to lipid accumulation and HNF-1α loss to altered PCSK9/LDLR expression, direct proof that rectifying HNF-1 function alone reverses lipotoxicity in established human-like CKD is still lacking.Third, the cell-type-specific functions of each HNF-1 isoform are poorly resolved. HNF-1α and HNF-1β have distinct renal distributions, implying they regulate different processes. A systemic drug targeting HNF-1 might therefore have opposing effects in various nephron segments, a critical complexity that current studies largely overlook.Fourth, from a therapeutic perspective, significant translational barriers exist. The development of isoform-selective and renal-specific HNF-1 agonists or antagonists is a formidable challenge, given the structural similarity between HNF-1α and HNF-1β and their widespread expression in vital organs. Off-target effects on hepatic, pancreatic, and intestinal metabolism could pose substantial clinical risks. These bottlenecks have seriously hindered the translation of mechanistic studies to the clinic.

As a pivotal regulatory node in the pathogenesis of lipid metabolic disorders, HNF-1 family has demonstrated growing translational potential in molecular diagnostics and targeted therapeutics. In order to overcome the current research bottleneck and elucidate the mechanistic roles of the HNF-1 family, it is necessary to build a multi-dimensional research framework: integrate single-cell sequencing and conditional gene editing technologies to analyze the regulatory network of HNF-1 family in specific cell types of kidney; leverage AI-driven platforms to predict dynamic interactions between HNF-1 family and upstream/downstream metabolic regulators; Design subtype-specific agonists coupled with renal-targeted delivery systems (nanoparticles, exosomes) to enhance intervention precision; and explore combined therapeutic strategies targeting HNF-1 family with both glucose-lipid crosstalk regulation and antifibrotic functions. Through interdisciplinary integration of multi-omics profiling, organoid disease modeling, and clinical cohort validation, these efforts will bridge the gap between mechanistic exploration and precision medicine for HNF1-associated renal pathologies.

## Data Availability

No datasets were generated or analysed during the current study.

## Abbreviations

3'UTR: 3' Untranslated Region
7-DHC: 7-Dehydrocholesterol
ABCA1: ATP-Binding Cassette Transporter A1
ACADL: Acyl-CoA Dehydrogenase Long-chain
Acat2: Acyl-CoA: Cholesterol Acyltransferase 2
ACC: Acetyl-CoA Carboxylase
ACE2: Angiotensin-Converting Enzyme 2
ACSL4: Acyl-CoA Synthetase Long-Chain Family Member 4
ADRP: Adipose Differentiation-Related Protein
ADTKD: Autosomal Dominant Tubulointerstitial Kidney Disease
AMPK: AMP-Activated Protein Kinase
ApoA1: Apolipoprotein A-1
ApoM: Apolipoprotein M
ASBT: Apical Sodium-Dependent Bile Acid Transporter
BMI: Body Mass Index
CAKUT: Congenital Anomalies of the Kidney and Urinary Tract
cAMP: Cyclic Adenosine Monophosphate; CBP: CREB-binding protein
ccRCC: Clear Cell Renal Cell Carcinoma
ChIP: Chromatin Immunoprecipitation
ChIP-seq: Chromatin Immunoprecipitation followed by Sequencing
ChRCC: Chromophobe Renal Cell Carcinoma
Chrebp: Carbohydrate Response Element-Binding Protein
CKD: Chronic kidney disease
co-IP: Co-Immunoprecipitation
CPT1α: Carnitine Palmitoyltransferase 1α
CREB1: CAMP Response Element-Binding Protein 1
CREB2: CAMP Response Element-Binding Protein 2
CRISPR: Clustered Regularly Interspaced Short Palindromic Repeats
C-TAD: C-terminal Transactivation Domain
CYP 2E1: Cytochrome P450 2E1
DCoH: Dimerization Co-factor of Hepatocyte Nuclear Factor 1
eGFR: Estimated Glomerular Filtration Rate
EMSA: Electrophoretic Mobility Shift Assay
ER: Endoplasmic Reticulum
ESRD: End-Stage Renal Disease
FAO: Fatty Acid β-Oxidation
FASN: Fatty Acid Synthase
FFA: Free Fatty Acids
FOXA3: Forkhead Box Protein A3
FXR: Farnesoid X Receptor
G6P: Glucose-6-Phosphate
G6Pase: Glucose-6-Phosphatase
GBD: Global Burden of Disease
GCKD: Glomerulocystic Kidney Disease
GLP-1: Glucagon-Like Peptide-1
GPX1: Glutathione Peroxidase 1
GPX4: Glutathione Peroxidase 4
GWAS: Genome-Wide Association Study
HDAC1: Histone Deacetylase 1
HDL: High-Density Lipoprotein
HMGCR: 3-Hydroxy-3-Methylglutaryl-CoA Reductase
HNF-1: Hepatocyte Nuclear Factor-1
HNF4α: Hepatocyte Nuclear Factor 4 Alpha
HSC: Hepatic Stellate Cell
KIM-1: Kidney Injury Molecule-1
LDL: Low-Density Lipoprotein
LDL-C: Low-Density Lipoprotein Cholesterol
LDLR: Low-Density Lipoprotein Receptor
LOX-1: Lectin-like Oxidized Low-Density Lipoprotein Receptor-1
Lp(a): Lipoprotein(a)
LXR: Liver X Receptor
MODY: Maturity-Onset Diabetes of the Young
MODY3: Maturity-Onset Diabetes of the Young type 3
MODY5: Maturity-Onset Diabetes of the Young type 5
NAFLD: Nonalcoholic Fatty Liver Disease
NaPi-IIa: Sodium-Phosphate Cotransporter Type IIa
NASH: Nonalcoholic Steatohepatitis
NEFA: Non-Esterified Fatty Acids
NGAL: Neutrophil Gelatinase-Associated Lipocalin
NOX4: NADPH Oxidase 4
NPC1L1: Niemann-Pick C1-Like 1
Nrf2: Nuclear Factor Erythroid 2-related Factor 2
OGTT: Oral Glucose Tolerance Test
p300: E1A-binding protein p300
p38 MAPK: P38 Mitogen-Activated Protein Kinase
PCB: Polychlorinated Biphenyls
PCK1: Phosphoenolpyruvate Carboxykinase 1
PCSK9: Proprotein Convertase Subtilisin/Kexin Type 9
PKA: Protein Kinase A
PKD: Polycystic Kidney Disease
POUH: POU homeodomain
POUS: POU-specific domain
PPARGC1A: Peroxisome Proliferator-Activated Receptor Gamma Coactivator 1-Alpha
PPARα: Peroxisome Proliferator-Activated Receptor Alpha
PPARγ: Peroxisome Proliferator-Activated Receptor Gamma
RCAD: Renal Cystic and Dysplastic Disease
RO: Renal Oncocytoma
ROS: Reactive Oxygen Species
RXR: Retinoid X Receptor
SCD-1: Stearoyl-CoA Desaturase-1
SGLT2: Sodium-Glucose Cotransporter 2
SNP: Single Nucleotide Polymorphism
SREBF2: Sterol Regulatory Element-Binding Transcription Factor 2
SREBP-1c: Sterol Regulatory Element-Binding Protein 1c
SREBP-2: Sterol Regulatory Element-Binding Protein 2
TC: Serum Total Cholesterol
TG: Triglyceride
VLDL: Very Low-Density Lipoprotein
